# How can corporate taxes contribute to sub-Saharan Africa’s Sustainable Development Goals (SDGs)? A case study of Vodafone

**DOI:** 10.1186/s12992-022-00894-6

**Published:** 2023-03-20

**Authors:** Eilish Hannah, Bernadette O’Hare, Marisol Lopez, Stuart Murray, Rachel Etter-Phoya, Stephen Hall, Michael Masiya

**Affiliations:** 1 The University of St Andrews, St Andrews, UK; 2 Zero Waste Scotland, Stirling UK and University of St Andrews, St Andrews, UK; 3 University of St Andrews, St Andrews UK and Bitwise Ltd, Dundee, UK; 4 Tax Justice Network and The University of St Andrews, St Andrews, UK; 5 University of Leicester, Leicester UK and University of Pretoria, Pretoria South Africa, Leicester, UK; 6 African Centre for Tax and Economic Studies (ACTES), Blantyre, Malawi

**Keywords:** Corporate taxes, Tax avoidance, Corporate social responsibility, Human rights, Right to health

## Abstract

**Background:**

The COVID-19 pandemic and the climate emergency threaten progress in reaching many of the Sustainable Development Goal (SDG) targets by 2030. The under-5 mortality and maternal mortality rates are well below the targets, and if we progress at the current pace, there is a high risk of not meeting the 2030 goals. Furthermore, the initial progress in the decline in child and maternal mortality since 1990 is likely to be eroded. Much of this progress has resulted from increased sanitation, drinking water, education, and health service coverage. The adequate provision of public services is possible if there is sufficient government funding. When governments have more income, they spend more on public services, which increases access to fundamental economic and social rights and, thus, contributes to the SDGs. One of the key drivers of government financing, taxation, constitutes 70% of government revenue in low- and lower-middle-income countries. Corporate income tax constitutes 18.8% of tax revenue in African countries compared to 10% of tax revenue in OECD countries. Therefore, it plays a critical role in SDG progress.

This paper aims to quantify the contribution of one large taxpayer, that publishes their tax payments, (Vodafone Group Plc) on progress towards SDGs in six African countries. We use econometric modelling to estimate the impact of an increase in government revenue equivalent to Vodafone's average tax paid between 2007–2017.

**Results:**

We find that government revenue equivalent to Vodafone’s taxes made a significant contribution to progress in attaining selected SDGs. We found that the revenue equivalent to Vodafone’s taxes allowed 966,188 people to access clean water and 1,371,972 people to access basic sanitation each year. Over the time period studied, 858,054 children spent an extra year in school and 54,275 children under five years and 3,655 mothers survived. In just one of these countries, Tanzania, the revenue equivalent to Vodafone's tax contribution allowed 174,121 people to access clean water and 223,586 to access sanitation each year. Over the time studied 187,023 children spent an additional year at school, 6,569 additional children under five and 625 additional mothers survived.

**Conclusions:**

These findings demonstrate that the reported contributions from a single multinational corporation drive SDG progress. Furthermore, it highlights the importance of transparent taxes and explores the responsibilities of global institutions, governments, investors, and multinational corporations.

## Background

### The right to health

The determinants of health (education, drinking water, and sanitation) are minimum core economic and social rights, which the United Nations Committee on Economic, Social and Cultural Rights have highlighted as the threshold below which no one should fall [1] (see appendix, Table 6). The African Charter on Human Rights (Article 16) also highlights the right of every individual to the best attainable state of health [2]. While different countries have varying abilities to provide for their citizens, these fundamental human rights are essential for human survival. They should be immediately accessible to everyone in every nation [3]. The Sustainable Development Goals (SDGs) are built on the foundations of human rights agreements, therefore we use the terms SDG progress and access to human rights interchangeably (see the appendix, Table 7, for definitions of the SDGs used in this analysis) [4]. Researchers have shown that much of the decline in child and maternal mortality since 1990 is due to increased coverage of water, sanitation and education (SDG 3,4 and 6), often provided as public services [5, 6].

SDG 3 aims to reduce the under-five mortality rate (U5M) to less than 25 per 1000 live births and the maternal mortality ratio (MMR) to less than 70 per 100,000 live births in all countries by 2030. However, in 2018, the U5M rate was, on average, 68 per 1000 live births across sub-Saharan African (SSA) countries (compared to 4.7 per 1000 live births in Europe) [7]. Equally staggering is the maternal mortality ratio, 547 per 100,000 live births in SSA (compared to 8 per 100,000 live births in Europe) [8]. Despite increases in survival rates in some regions, many countries are unlikely to meet the SDG targets for maternal or child mortality by the target year of 2030, and many of the targets are further threatened by the COVID-19 pandemic and the climate emergency [9].

There are many reasons a government may not provide critical services, and a lack of tax revenue is prominent among them [10]. Therefore, increased government revenue resulting from progressive taxation is the most sustainable strategy to ensure governments fulfil their human rights obligations and provide services essential for health [11].

Fair taxes have been described as paying the right amount of tax (but no more) at the right time and place according to the letter and spirit of the law and providing sufficient public information for external critique [12]. Taxes are necessary to allow governments to have the fiscal capacity to provide public services and should, at the minimum, be ‘fair’. Domestic and multinational businesses are the primary drivers of economic growth and job creation, and their tax contributions are vital to domestic resource mobilisation and public spending [13]. Given the private sector’s pivotal role in most economies, profit must be taxed fairly. This also enables a country to provide a conducive environment to attract foreign direct investment. For companies that operate across borders, often called multinational corporations (MNCs), it is critical that they pay taxes where real economic activity takes place [14]. On the other hand, tax abuse (defined as tax avoidance or tax evasion, see Table 1) [15] is not uncommonly used to avoid a fair share of the tax burden and increase profits [16]. Despite growing criticism in the media and attention by advocacy groups, some entrepreneurs consider tax avoidance and tax planning integral to modern business practice, contradicting many corporations' avowed social responsibility aspirations [17, 18].

**Table 1:** Tax term definitions

Tax avoidance	Generally used to describe the arrangement of a taxpayer’s affairs intended to reduce their tax liability. Although the arrangement could be strictly legal, it usually contradicts the intent the law purports to follow [19]
Tax evasion	Generally means illegal arrangements where liability to tax is hidden or ignored, i.e. the taxpayer pays less tax than they are legally obligated to pay by hiding income or information from the tax authorities [19]
Tax Abuse	Tax avoidance and tax evasion [20]

The seventeen SDGs are the centrepiece of the 2030 Agenda for Sustainable Development, grounded in international human rights and are a plan to build an equitable and sustainable world [21, 22]. The overarching agenda for financing the SDGs emphasises the importance of domestic resource mobilisation, and one of the targets of SDG 16 is to reduce tax abuse, while SDG 17 aims to support domestic resource mobilisation [23].

We demonstrate how corporate tax payments accelerate progress towards the SDGs by studying the contributions of one MNC, a telecommunications company. We selected Vodafone Group PLC, hereafter referred to as Vodafone, because they publish their taxes on a country-by-country basis, and they state that they do not artificially transfer profits from one jurisdiction to another to minimise tax payments, and the accounts are independently audited.

### Financing the sustainable development goals

Reeves et al. found that increased tax revenue was associated with increased government health spending, while Baldacci showed that increasing government spending on health and education increased child survival [24, 25]. Moreover, governments with robust revenue streams are more likely to allocate resources to critical services [26], and increased revenue across all sectors is vital to ensuring human rights obligations and SDG progress.

Governments principally use tax revenue to fund public services. There are two steps from taxes to the SDGs. The first step is to raise revenue, and the second is to allocate it (with some governments earmarking revenue for specific SDGs). If income is redistributed to support the SDGs, tax revenue can be said to have supported progress [27]. To enhance the role of tax in financing the SDGs, experts have developed frameworks to support governments in aligning their tax policies and assessing corporations' broader contribution to the SDGs [28].

Taxes make up, on average, 33.8% of GDP in OECD countries compared to 16.6% in African countries and account for around 85% of total government revenue in high-income and 71% of government revenue in low- and lower-middle (hereafter lower-) income countries [29]. However, the average share of corporate income tax revenues in total tax revenues in Africa was 18.8%, compared to 10% in OECD countries; therefore, it plays a significant role in raising vital revenue for human rights and development in lower-income countries [30].

Tax revenue gaps threaten the effective contribution of tax revenue to the government budget. These gaps can arise both domestically and internationally [10]. Reducing the domestic tax gap includes reviewing tax policies and strengthening the efficiency of tax administrations to detect and deter revenue leakages. For example, many lower-income countries introduced value-added tax (VAT) over the last few decades, and this was very effective in raising revenues (although opinions vary about its merit in the tax policy mix) [31]. Other tax policies include reducing tax expenditures (tax incentives and exemptions), increasing taxes on wealthy individuals, and integrating the informal sector into the formal economy. The success of these initiatives is dependent on the efficiency of tax administrations. However, experts do not anticipate that domestic tax policy choices will significantly reduce the domestic tax gap in the short term [29]. Whilst it is vital to address these areas, the focus of this paper is on corporation tax.

### The role of corporate taxes in SDG progress

Corporate tax is critical in countries with minimal opportunities to reduce the domestic tax gap, where developmental needs are vast and immediate. For countries requiring revenue to reach the SDGs, narrowing the international tax gap may represent the most viable source of additional funding in the short to medium term [32]. In addition, surveys show that most businesses are familiar with the SDGs and plan to incorporate them into their business practices [33]. Therefore, ensuring a fair tax strategy would be in line with this.

Any additional corporate tax could play a critical role in lower-income countries as the relationship between government revenue per capita and progress toward the SDGs is highly non-linear, and small increases can have large effects, when government revenue per capita is small [34, 35]. In addition, an empirical study by Gaspar et al. identified a tax to Gross domestic product (GDP) tipping point. A tipping point is when small changes give rise to significant outcomes, and they estimate that when the tax to GDP ratio is 12.75%, the real GDP increases sharply and sustainably over the next decade [36]. Thus, corporate tax could play a crucial role in increasing  the tax to GDP ratio in lower-income-countries and, consequently, real economic growth. Unfortunately, international corporate tax avoidance and tax evasion deprive governments of vital revenue required to achieve the SDGs [16].

Robust revenue streams are necessary to ensure efficient governments and institutions and facilitating good governance. Indeed, it has been empirically shown that an increase in revenue drives an improvement in governance over time in all countries [37]. An empirical study of 23 sub-Saharan African countries demonstrated that increasing fiscal capacity through increasing the tax-to-GDP ratio leads to improved governance and reduced corruption. The pathway between increased revenue and improved governance acts by strengthening the fiscal contract between the state and citizens, as opposed to the ability to provide better remuneration for civil servants [38]. Therefore, small increases in corporate tax revenue could play a pivotal role in tipping the balance towards SDG progress in some countries. Increased government revenue from corporate taxes is a sustainable strategy to ensure that governments fulfil their human rights obligations and provide services essential for health [11].

### The role of corporate social responsibility

The idea that business enterprises are responsible to society beyond profit is not new, but it has received more attention over the last few decades as excessive profits have raised concerns that companies prioritise shareholders over other societal stakeholders [39]. There is an ongoing debate about what corporate social responsibility (CSR) is, what it achieves, and what it could achieve. Generally, it covers a company’s responsibilities to society and the environment where they operate. It incorporates these needs into its decision-making, as the sole principle of maximising shareholder wealth may not benefit all stakeholders [40]. The broad reasons for CSR engagement include moral responsibility, sustainability, regulations, and reputation. One study examined three MNCs which experienced scandals with reputational impact. The researchers found that pre-emptively incorporating CSR considerations into their supply chains would have resulted in a competitive advantage in the long run [41].

However, CSR activities are often ad-hoc with little social impact and are in response to society's expectations that companies are good global citizens [40]. Some argue that CSR has historically and categorically failed to create positive social change because communities are not determining the priorities and, in some cases, CSR is undertaken to mask the harmful effects of MNCs with highly visible impacts through small projects at the micro-level. In addition, it improves reputation and, thus, the relationship with broader society; doing so may create a financial return, but this is debatable. As economic inequality is rising, Visser recommends that businesses move to radical CSR. Radical CSR calls for changes to the systems that underpin capitalism as we know it and taking steps to ensure that the world conducts business to benefit global society, rather than a select few, thus avoiding grievous social, economic, and environmental harm [42].

While some argue that businesses should develop CSR standards on Taxation [43], others say that fair tax is integral for any law-abiding MNC and outside the CSR framework because CSR is discretionary.

### Vodafone and tax controversies

Vodafone is a publicly listed telecommunications company on the UK and the US stock exchange. It was established in 1991 and is one of the UK’s largest and most successful companies, it employs 94,000 people and has subsidiaries in 47 countries. Vodafone Global Enterprise provides telecommunications services to clients in 168 countries [44].

In 2010, Private Eye, a British satirical current affairs magazine, reported that Vodafone’s acquisition of a German company was routed through a Luxembourg subsidiary to avoid paying tax in the UK. This controversy led to widespread protests and shop closures across the UK. A subsequent deal with Her Majesty’s Revenue and Customs (HMRC) agreed that Vodafone would pay £1.25 billion, the case was settled because HMRC may have lost in court. However, some estimate the company avoided paying £6 billion of tax in the UK [45]. Vodafone’s tax report from 2012 addresses this issue, stating that it was a complex interpretation of a UK law, and the European Court of Justice, the UK High Court and the UK Court of Appeal reviewed the case before settling.

Some have criticised Vodafone for paying less than their fair share of corporation tax in the UK [46]. The 2012 report addresses this, it is states that  "In a number of Vodafone's markets, including the UK, the cost of acquiring radio spectrum from the government, high operating costs, substantial levels of capital expenditure and sustained competitive and regulatory pressures have a significantly negative effect on the profits of our local businesses" [47].

In 2007, Vodafone acquired a company in India, and there was a dispute with the Indian government over capital gains tax. A committee subsequently ruled in favour of Vodafone that capital gains tax should be paid by the seller and not the buyer [45].

### Aim

We aim to estimate the increase in the number of people who would access their rights due to the tax contributions of Vodafone in six African countries (the Democratic Republic of Congo (DRC), Ghana, Kenya, Lesotho, Mozambique, and Tanzania). We focused on Africa because it is the continent with the highest child and maternal mortality and the smallest proportion of people accessing their fundamental rights.

This paper aims to quantify the private sector's contribution toward the SDGs and move fair tax further up MNCs' and institutional investors' agendas. We selected Vodafone as a case study because public tax reports are available country-by-country. We then highlight key areas globally and nationally that need to be addressed to ensure corporate tax abuse does not harm human rights.

While previous studies have highlighted the harms tax abuse can cause to human rights, to our knowledge, this is the first study to quantify the private sector’s contribution to the SDGs [48, 49]. Thus, we highlight the vast potential should all business enterprises reflect on their tax policies. In an age where CSR and a stakeholder practice model are increasingly important, similar modelling offers opportunities for both small companies and MNCs to quantify and demonstrate their commitments to the SDGs and human rights.

## Methodology

### The model used to quantify Vodafone’s contribution to public finances

We employed economic modelling from the Government Revenue and Development Estimations (GRADE) tool (v3.3.0, 2022/09/08) to estimate the increase in the number of citizens accessing their rights when there is an increase in government revenue equivalent to Vodafone's reported tax contributions [50, 51]. The GRADE uses data from countries worldwide to model the impact of government revenue and governance on the coverage of the SDGs, access to water, education, and sanitation, and maternal and child mortality. The GRADE is available as an online visualisation [51], and demonstrates that even a small increase in government revenue has a massive impact in lower-income countries. The model is precise, as shown by comparing the modelled and the actual coverage of the determinants of health, which are generally within one percentage point, and available in Mendeley Datasets [52]. As well as being precise, the GRADE modelling is realistic as it assumes that any additional government revenue will be spent in the same way as it has been in the past and therefore avoids the incorrect assumption that governments will allocate all additional income to one specific sector. For example, if a government typically allocates 10% to health spending and receives additional revenue, then 10% of the additional revenue would be allocated to health. Furthermore, the benefits of an increase in government revenue takes at least five years to become apparent, and the model incorporates this lag effect. Downstream outcomes such as survival are influenced by government spending on all sectors, for example, infrastructure and agriculture, and GRADE modelling incorporates these broader impacts. Hence, the model provides a robust estimation of the effects of government revenue on the SDGs and the impact of an increase in revenue on governance. The GRADE uses government revenue (excluding grants and including social contributions) from the UNU WIDER Government revenue database and the GDP in 2015 constant US dollars taken from the World Development Indicators [53, 54]. The modelling includes six dimensions of quality of governance from the World Governance Indicators (see Table 8 in the appendix for the definitions) [55].

Vodafone’s reported contributions to public finances in six countries – Democratic Republic of Congo (DRC), Ghana, Kenya, Lesotho, Mozambique, and Tanzania – were analysed. Vodafone has published its contributions to governments in their 'Taxation and our total economic contribution to public finances' reports from 2012–2018 [47, 56–60]. Contributions include direct revenue, other direct non-taxation, and indirect revenue contributions [61]. The total tax contribution for each country and year was converted into United States Dollars (USD) using the nominal exchange rate listed in each report. The total tax contribution was converted into 2015 USD, the base year used in the GRADE. Tax contributions fluctuate each year; therefore, we used the average for the seven years. The latest year with data for maternal mortality is 2017, thus, the period analysed was 2007 – 2017. We assumed that Vodafone contributed the average calculated for 2012–2018 between 2007–2017, and the maximum benefit was accrued after five years. (An alternative approach would be to use the average contribution as a percent of government revenue).

For illustration purposes, Table 2 shows Vodafone’s total contributions to governments in 2018 from their annual report [61]. The total contributions are the sum of columns c, d and e (direct revenue contribution tax, direct revenue contribution non-tax and indirect revenue contributions, see Table 2 for definitions). Column j shows this as a percentage of government revenue. Table 3 shows the total contribution to public finances per country between 2012–2018, and column h shows the average.

**Table 2:** Total contribution to public finances 2018 in six African countries

	**Revenue (a)**	**Profit before taxes (b)**	**Direct revenue contributions: taxation mechanisms (c)**			**Direct revenue contribution: non-taxation mechanisms (d)**	**Indirect revenue contribution: Indirect taxes (e)**	**Capital investment (f)**	**Direct employment (g)**	**Total contribution (h)**			**Government revenue excluding grants including social contributions 2018** $M **USD 2015 (i)**	**Vodafone’s contribution as a percentage of Government revenue (j)**
			Total	*Split between*										
				Direct taxes	Corporate tax									
	FY17–18 €m	FY17–18 €m	FY17–18 €m	€m	€m	FY17–18 €m	FY17–18 €m	FY17–18 €m	FY17–18 €m	Euro €m	USD $M 2018	USD $M 2015		
DRC	359	(65)	18	17	2	27	77	45	599	122	143	135.70	3742071992	3.6
Ghana	253	(168)	19	7	12	9	55	33	1,052	83	97.11	91.97	9132934260	1.0
Kenya	781	370	308	102	206	19	99	121	1,761	426	498	472.02	13130469537	3.6
Lesotho	72	31	8	< 1	8	4	8	10	206	20	23.40	22.16	909597717.7	2.4
Mozambique	231	68	15	3	12	6	26	58	512	47	54.99	52.08	4136194745	1.3
Tanzania	370	40	26	9	17	9	122	61	530	157	184	173.96	7930527182	2.2

^a^Total revenue

^b^Total taxable revenue in each country minus allowable expenses

^c^This includes corporation tax, business rates or equivalent, employers’ national insurance contributions or equivalent, sector-specific taxes (such as ‘special’ taxes or ‘telecoms’ taxes) and other taxes

^d^Other forms of revenue raised by the government and a country’s direct taxation regime, including telecoms licence fees

^e^Taxes collected on governments’ behalf, including pay as you earn (PAYE) income tax, employees’ national insurance contributions, withholding taxes, sales and consumption taxes and value-added tax (VAT)

^f^Investments in building and maintaining the networks and services relied upon by the 700 million mobile and 21 million broadband customers

^g^The average number of people employed in the 2018 financial year. This includes direct employees and the relevant share of employees who work for our joint ventures, associates, or other part-owned companies

^h^Total contributions to governments (total of column c, d and e)

^i^Total government revenue

^j^Percentage contribution to government revenue (h)/(i)

**Table 3:** Total contribution to public finances per country each year between 2012- 2017

Country	2012 in 2015 $m (a)	2013 in 2015 $m (b)	2014 in 2015 $m (c)	2015 in 2015 $m (d)	2016 in 2015 $m (e)	2017 in 2015 $m (f)	2018 in 2015 $m (g)	Average yearly contribution in 2015 $m (h)
DRC	103.8	103.98	120.4	135.25	161.4	137.85	135.18	128.27
Ghana	90.39	76.36	83.48	75.67	79.20	91.9	92.00	84.14
Kenya	366.63	172.22	231.16	264.06	276.47	356.91	472.02	305.64
Lesotho	16.74	13	11.24	9.66	13.45	18.17	22.17	14.91
Mozambique	6.7	8.12	12.85	28.98	34.37	50.22	52.08	27.62
Tanzania	80.4	146.22	197.45	191.61	156.91	167.77	173.96	159.19

## Results

We found that the government revenue equivalent to Vodafone's average contribution ensured 966,188 people access clean water and 1,371,972 accessed sanitation each year. Over the time studied  these contributions enabled 858,054 children to spend an additional year in school. As a result of increased access to their determinants of health, 54,275 additional children under five and 3,655 additional mothers survived, see Table 4. We report the increase in access to rights or SDG progress in six SSA countries because of increased government revenue equivalent to Vodafone's tax contributions in Table 5.

**Table 4:** Summary of the SDG progress and access to fundamental rights associated with increased government revenue equivalent to Vodafone's contribution to public finances

Variable	Numbers with increased access
Access to basic drinking water (each year)	**966, 188**
Access to basic sanitation (each year)	**1, 371, 972**
Additional years in school (cumulative over the time studied)	**858, 054**
Child deaths averted (cumulative over the time studied)	**54, 275**
Maternal deaths averted (cumulative over the time studied)	**3, 655**

**Table 5:** SDG progress and  access to fundamental rights associated with increased government revenue equivalent to Vodafone's contribution to public finances in six countries

	**The average increase in government revenue equivalent to Vodafone's contribution $ million**	**Individuals with increased access to basic drinking water each year**	**Individuals with increased access to basic sanitation each year**	**Children who attend school for an additional year (over the time studied)**	**Child deaths averted (over the time studied)**	**Maternal deaths averted (over the time studied)**
DRC	**128.27**	**335, 749**	**195, 752**	**558, 964**^**a**^	**36, 910**	**1, 677**
Ghana	**84.14**	**115, 442**	**143, 994**	**38, 763**	**1, 645**	**200**
Kenya	**305.64**	**295, 257**	**749, 492**	**26, 527**^**b**^	**7, 163**	**942**
Lesotho	**14.91**	**4, 590**	**4, 293**	**1, 696**	**150**	**16**
Mozambique	**27.62**	**41, 029**	**54, 855**	**45, 081**	**1, 838**	**195**
Tanzania	**159.19**	**174, 121**	**223, 586**	**187, 023**	**6, 569**	**625**
Totals	**Na**	**966, 188**	**1, 371, 972**	**858, 054**	**54, 275**	**3, 655**

^a^Data is only available up to 2015

^b^Data is only available up to 2009

## Discussion

We demonstrate that government revenue equivalent to the tax contribution of just one MNC is associated with significant increases in access to the determinants of health (i.e., drinking water, sanitation, and education), which are fundamental rights, in six countries. Thus, we demonstrate how Vodafone has contributed towards SDG progress in six host countries. The benefits include almost a million people accessing clean water and over a million accessing basic sanitation, each year. Over the study period more than 800,000 children spent an additional year in school, over 54,000 children survived and almost 4,000 mothers survived. These figures demonstrate MNCs’ substantial impact for several reasons: Firstly, government revenue in lower-income countries is small, and any additional income will be relatively large. As shown in Table 2, the tax contributions from Vodafone alone accounted for 1–3.6% of government revenue in 2018 in these six countries, whereas in the UK, Vodafone's contribution accounted for 0.16% [57, 62]. Secondly, interventions that substantially reduce mortality in lower-income countries include public health measures such as clean water, sanitation, education and primary health care, which are less costly than interventions required to reduce mortality in high-income countries [63].

Thus, corporate tax contributions contribute to SDG progress in lower-income countries. Corporations, governments, consumers, investors, and international organisations play a role in SDG progress by promoting fair corporation tax. We discuss these below [62].

### Limitations

We do not have access to the previous tax year reports for 2007, 2008, 2009, 2010, and 2011. Therefore, we have assumed the contribution for these years was the same as the average. Equally, the reported revenues were not analysed for misalignment. Misalignments are inconsistencies between reported profit and actual economic activity [64]. We did not have data on maternal mortality past 2017, so we could not analyse a later study period. We did not have education data past 2009 in Kenya, meaning the full benefit of education will be underestimated. In addition, we did not have data on education for the DRC past 2015.

### Multinational corporations and the sustainable development goals

While governments are crucial to steering SDG progress, businesses are vital contributors to public finances and can positively or negatively impact progress with their policies and practices. Indeed, Barnett argues that all law-abiding MNC activities have a social component because they improve the economic conditions of society [65]. Many companies engage with the SDGs, but Oxfam suggests that before business enterprises try to do good, they should first not harm by reviewing their supply chains, employment policies, and tax planning arrangements [66]. A review of corporate governance and tax avoidance literature finds that most firms pay above the average statutory rate and resist opportunities to reduce their tax burden. In contrast, a few aggressively avoid tax [67].

Corporate tax abuse erodes access to rights including the right to life. Business enterprises must not undermine a state's ability to meet their human rights obligations, especially as it may be easier to avoid and evade tax where host country governance is poor, which is precisely where the tax revenue is most needed and indeed would improve governance. Tax abuse not only erodes economic and social rights as a result of foregone revenue, but also the right to an effective government [37]. Moreover, activities to support rights locally, for example, a clinic or school, while laudable, do not offset a failure to promote rights and good governance nationally by paying taxes.

Given the legal and ethical controversies surrounding tax abuse, the International Bar Association's Human Rights Institute (IBAHRI) has made critical recommendations [16]. It recommends that business enterprises adopt and commit to human rights throughout all operations, including due diligence measures and impact assessments on tax planning practices, financial flows and tax revenues generated in different jurisdictions. It advises against negotiating special tax holidays, incentives and rates that prevent governments from fulfilling their human rights obligations and promote transparency through public reporting country-by-country.

Certain aspects of corporate governance reduce tax abuse, such as robust governance structures, an independent audit committee, and separation between ownership and management, as in publicly traded companies. Drivers of abuse include an incentive structure based on after-tax profits that induce risk-taking by those who benefit. Indeed, individuals in crucial positions may drive tax abuses in whichever firm they work for [67]. Media coverage of tax abuse and the subsequent introduction of stronger taxation laws, including the OECD's two-pillar approach [68], has increased scrutiny of MNCs' tax practices, see the Global Governance section.

### Tax and corporate social responsibility

Society needs successful businesses, domestic and MNCs. Enterprise requires a healthy and educated population, which requires critical services that rely on robust infrastructure and institutions which need tax revenues [40]. However, there is a perception and evidence that some corporations avoid taxes, which has led some to propose that taxes should be an essential component of CSR strategies [43, 69]. In contrast, others, notably Milton Freedman, argue that corporations are only responsible for their employees (shareholders or proprietors). They must conduct business with this in mind while conforming to society’s laws, including ethical customs. In his thinking, if an executive chooses to spend shareholder money on social goods, they are spending money that is not their own and on sectors where they have no expertise. He states that the imposition of taxes and determining the spending of this revenue is the function of the government, not business [70].

Tax avoidance increases profits for shareholders in the short term and increases executive bonuses (if based on after-tax profits). However, the long-term impact may be harmful, with the risks including reputational damage and litigation. Empirical studies show that businesses that may engage with CSR hedge against negative public opinion if tax abuses become public [71]. There is an association between companies that rank highly on CSR indexes and corporate tax abuse among companies listed on the Chinese stock exchange. This finding supports the view that CSR may be a substitute for tax payments [72]. These findings are aligned with the school of thought that it is possible to compensate for tax abuse with CSR or that tax abuse is justified to pay CSR expenses [73]. There is also a question of sovereignty and national development policy and planning. Without consultation with the state, companies may decide on CSR strategies that are not national development priorities and are may have unintended consequences. Nonetheless, the relationship between CSR and corporate tax avoidance varies; for example, a study in Australia finds that companies which engage in CSR are less likely to engage in tax abuse [74].

We agree that it is a government's, not a business's, responsibility to redistribute tax revenue and use them to respect, protect and fulfil human rights and SDG progress and fair tax does this [75]. The private sector's role is to support governments in meeting their obligations by paying their fair share of tax and this should be outside the CSR framework. Therefore, we agree with Oxfam that businesses should first not harm by ensuring tax transparency and fair tax payments before doing good with CSR activities.

Businesses have started to publish regular CSR reports; for example, of the 500 largest MNCs listed on the USS stock exchange, only 11% were posted in 2011 compared to 85% in 2017 [76]. However, in an era where the international community is calling for MNCs to combat rising global inequality, brands have trouble using CSR to stand out from the crowd. Therefore, cutting-edge methods are required to increase brand value [77].

Fair and transparent tax practices can demonstrate how an MNC tackles global inequality. A financial incentive exists because companies' stock market prices fall when tax abuse is made public [78]. In addition, boycotts have included Starbucks in the UK and Burger King in the US, related to tax abuse scandals [79]. Scrutiny by the public and protests have moved tax issues up the agenda to the boardroom [80]. In addition, 68% of participants of a Dutch pension fund preferred their pension fund managers to invest responsibly, even if this resulted in lower returns [81].

### Investor’s responsibilities

Investors increasingly incorporate CSR into portfolio decisions as responsible and sustainable investing increases in popularity. For example, the United Nations Secretary-General convened an extensive global network of institutional investors to develop the Principles for Responsible Investment (PRI). The PRI signatories publicly commit to incorporating CSR issues into investment analysis and decision-making, pursue standardised reporting, and encourage all investors to adopt the principles [82]. Signatories to the PRI had $80 trillion of assets under management in 2019. The three most prominent institutional investors (Blackrock, State Street Global Advisors, and the Vanguard Group) are signatories.

Long-term institutional investors are more risk-averse and may guide their investees towards tax compliance [67]. Investors’ fiduciary duties require that they invest prudently and, in their client’s, best interest. Integrating fair tax into investment strategies depends on whether the investor believes these will materially affect the portfolio's performance. We believe investors can massively support SDG progress by encouraging all MNCs to adopt fair and transparent tax practices. As potentially large shareholders that engage regularly with companies they have comparative advantage to perpetuate real world change [83].

### Host country responsibilities

According to international human rights law, countries must respect, protect, and fulfil human rights within their territory and jurisdiction and are obligated to use all available tools and resources for this purpose. This duty includes safeguarding their citizens and business enterprises against infringements by other actors. Tools include legislation, policies, regulations and adjudication, which should be anchored in the constitution [26]. Equally, governments must request the right amount of tax, but no more, to further develop the vital infrastructure that businesses and citizens need to thrive.

In 2020, a High-Level Panel on Financial Transparency, Accountability, and Integrity (FACTI) was convened to strengthen integrity within the global financial system. The FACTI panel report was published in 2021 [84]. Tax abuse is a worldwide challenge, but actions at the national and African levels complement global efforts. Measures which countries have taken or could take to address tax abuses include enacting legislation to mandate automatic exchange of information between tax authorities, beneficial ownership of companies and country-by-country reporting. We explore these concepts further in the global governance section. However, while 31 of the 54 African Union members have legislated for automatic exchange of information, only 14 have a beneficial ownership law and nine a law for country-by-country reporting [85].

Governments may need to invest in the revenue authorities and review tax incentives and treaties to counter tax abuse and maximise public finances. Every country that receives overseas development aid should invest in its revenue authorities to decrease its dependence on aid [86]. Governments try to balance the need to provide an attractive environment for corporations with ensuring that all large taxpayers contribute to the public purse. This is complicated by competition for the same foreign investment and the resulting pressure to use tax incentives or waivers to attract investment. However, incentives reduce the amount of corporate tax revenue and drive a race to the bottom.

### Home countries of MNCs

Countries that facilitate tax abuse violate their international human rights obligations. General comment number 24 (regarding extraterritorial obligations in the context of business activities) declares that they are required to take steps to prevent human rights violations abroad by corporations [87]. Some countries bear more responsibility for tax abuses than others [15]. The IBAHRI highlights the damaging impact of tax abuse, and those obligations include 'doing no harm' to economic, social, and cultural rights abroad. They highlight the key areas conducive to tax abuses. These include transfer pricing and other cross-border intra-group transactions, the negotiation of tax holidays and incentives; the taxation of natural resources; and offshore investment accounts. Secrecy jurisdictions or tax havens and enablers (accountants and lawyers) cost governments $500–600 billion annually because of their role in facilitating tax abuses [88, 89]. Home countries should consider the obligation to 'do no harm' to rights to include an obligation for states to assess and address corporate tax policies' domestic and international impacts. If a business enterprise receives state support, for example, an export credit guarantee, there is an additional onus on the home country to ensure that the supported business does not engage in tax abuse. Additionally, the promotion of transparency as well as technical assistance for lower-income countries to increase their domestic revenue capacity will become essential to future development agendas [16].

### Global governance

Collectively, states are the trustees of the international human rights regime and collective action through multilateral institutions could play a critical role in the field of tax. While the gap in global governance regarding taxation is significant [90], there are recommendations and initiatives to address these. Tax justice advocates recommend that all countries should adopt three measures known by the 'ABC acronym': automatic exchange of information, beneficial ownership registration and country-by-country reporting. Automatic exchange of information means countries automatically share relevant financial information on corporations and individuals; this makes it easier for other jurisdictions to trace illicit finance. Beneficial ownership is the practice of registering the person or people who own a company. Country-by-country reporting refers to the practice of publishing profits made and tax paid in each country where the corporation operates. A UN tax convention would be key to oversee these reforms and all countries should actively support this [91]. Progress was made under the G20/OECD project now known as the BEPS framework, but advocacy groups argue that lower-income countries were negatively impacted by the deal and excluded from decision-making. A game changing proposal for a UN tax framework by the Africa Group was approved at the UN by consensus at the 77th session [92]. Human rights experts and committees have been highlighting the negative effects of cross border tax abuse on the realisation of human rights for more than a decade [91]. For example, the United Committee on the Rights of the Child (UNCRC) has requested that Ireland and the Netherlands review their corporate tax policies to ensure they do not harm child rights abroad [86].

## Conclusion

We demonstrate that one MNC, through contributing to public finances, has a positive impact on access to fundamental rights, progress to the SDGs, and ultimately survival in six African countries. This case study adds weight to the argument for fair tax. Other MNCs could use the GRADE to demonstrate their contribution to SDG progress through taxes paid. Fair taxation is vital for any MNC and business enterprise to support home and host countries to meet their human rights obligations. We believe that fair tax should be prioritised before any CSR activity. Equally, investors can play a pivotal role in supporting MNCs to adopt fair and transparent tax policies; it is not just a governance issue but a human rights issue.

This study adds to growing evidence that tax is a human rights issue and critical for the SDGs. This is the first study to quantify one MNC's contribution to SDG progress. We have summarised key global and national policies and responsibilities that must be addressed to drive fair tax policies. We plan to study other MNCs that publish their contribution to public finances to highlight their role in SDG progress and respect for human rights. We will use this evidence to advocate for global action on tax abuse.

## Data Availability

Data supporting these results can be found in Vodafone's 'tax and economic contribution reports' and Vodafone's annual reports on their website. Data for the GRADE modelling was obtained from the UNU WIDER Government revenue database and the world bank, which is also available publicly. Papers explaining the modelling can be obtained via the GRADE website https://med.st-andrews.ac.uk/grade/.

## Appendix

Tables 6, 7 and 8.

**Table 6:** Minimum core obligations ( Office of the United Nations High Commissioner for Human Rights, 2008)

Ensure the right of access to employment, especially for disadvantaged and marginalised individuals and groups, enabling them to live a life of dignity
Ensure access to the minimum essential food that is nutritionally adequate and safe, to ensure freedom from hunger to everyone
Ensure access to basic shelter, housing, and sanitation, and an adequate supply of safe drinking water
Provide essential drugs as defined under the World Health Organization's Action Programme on Essential Drugs
Ensure free and compulsory primary education for all
Ensure access to a social security scheme that provides a minimum essential level of benefits that cover at least essential health care, basic shelter and housing, water and sanitation, food, and the most basic forms of education

**Table 7:** The Sustainable development goals and indicators used in this study (96)

SDG 3—Good health and well-being Ensure healthy lives and promote well-being for all at all ages Targets: • By 2030, reduce the global maternal mortality ratio to less than 70 per 100,000 live births • By 2030, end preventable deaths of new-borns and children under five years of age, with all countries aiming to reduce neonatal mortality to at least as low as 12 per 1,000 live births and under-5 mortality to at least as low as 25 per 1,000 live births *Indicators used in this study: Child and maternal mortality rates*
SDG 4 – Quality education Ensure inclusive and equitable quality education and promote lifelong learning opportunities for all Targets: • By 2030, ensure that all girls and boys complete free, equitable and quality primary and secondary education leading to relevant and effective learning outcomes *Indicators used in this study: Additional school years*^*a*^
SDG 6 – Clean water and sanitation Ensure availability and sustainable management of water and sanitation for all Targets: • By 2030, achieve universal and equitable access to safe and affordable drinking water for all • By 2030, achieve access to adequate and equitable sanitation and hygiene for all and end open defecation, paying special attention to the needs of women and girls and those in vulnerable situations *Indicators used in this study: Access to drinking water and sanitation*^*a*^*, *^*b*^

^a^See appendix for further information

^b^We differentiate between basic and safe drinking water and sanitation

## Definitions

Basic drinking water services – the percentage of the population drinking water from an improved source, provided collection time is not more than 30 min for a round trip. This indicator encompasses both people using basic water services as well as those using safely managed water services. Improved water sources include piped water, boreholes or tube wells, protected dug wells, protected springs, and packaged or delivered water.

Basic sanitation services—the percentage of the population using at least, that is, improved sanitation facilities that are not shared with other households. This indicator encompasses both people using basic sanitation services as well as those using safely managed sanitation services. Improved sanitation facilities include flush/pour flush to piped sewer systems, septic tanks or pit latrines, ventilated improved pit latrines, compositing toilets or pit latrines with slabs.

School life expectancy (primary and secondary), both sexes (years)—the number of years a person of school entrance age can expect to spend within the specified education level. For a child of a certain age, the school life expectancy is calculated as the sum of the age-specific enrolment rates for the levels of education specified. The part of the enrolment that is not distributed by age is divided by the school-age population for the level of education they are enrolled in and multiplied by the duration of that level of education. The result is then added to the sum of the age-specific enrolment rates. A relatively high SLE indicates a greater probability of children spending more years in education and higher overall retention within the education system. The expected number of years does not necessarily coincide with the expected number of education grades completed because of repetition. Since school life expectancy is an average based on participation in different levels of education, the expected number of years of schooling may be pulled down by the magnitude of children who never go to school. Those children in school may benefit from many more years of education than the average. Here education is shown as the percentage of the maximum SLE (primary and secondary), both sexes (years), globally, which is 17 years.

**Table 8:** Definitions of quality of governance from the world governance indicators [56]

Governance Dimensions	What it captures
Control of corruption	Perceptions of the extent to which public power is exercised for private gain, including both petty and grand forms of corruption, as well as 'capture' of the state by elites and private interests
Government effectiveness	Perceptions of the quality of public services, the quality of the civil service and the degree of its independence from political pressures, the quality of policy formulation and implementation, and the credibility of the government's commitment to such policies
Political stability	Perceptions of the likelihood that the government will be destabilised or overthrown by unconstitutional or violent means, including politically motivated violence and terrorism
Regulatory quality	Perceptions of the ability of the government to formulate and implement sound policies and regulations that permit and promote private sector development
Rule of law	Perceptions of the extent to which agents have confidence in and abide by the rules of society, and in particular the quality of contract enforcement, property rights, the police, and the courts, as well as the likelihood of crime and violence
Voice and accountability	Perceptions of the extent to which a country's citizens can participate in selecting their government, as well as freedom of expression, freedom of association, and a free media

## Abbreviations

SDG: Sustainable development goals
DRC: Democratic Republic of Congo
U5M: Under-five mortality rate
MMR: Maternal mortality ratio
SSA: Sub-Saharan Africa
VAT: Value-added tax
GDP: Gross domestic product
MNC: Multinational corporation
CSR: Corporate social responsibility
HMRC: Her Majesty's Revenue and Customs
GRADE: Government Revenue and Development Estimations
IBAHRI: International Bar Association's Human Rights Institute
PRI: Principles for Responsible Investment
UNCRC: United Nations Committee on the Rights of the Child
EITI: Extractive industries transparency initiative

## References

[1] Office of the United Nations High Commissioner for Human Rights (2008). The Human Rights Fact Sheet 33: Frequently Asked Questions on Economic, Social and Cultural Rights.

[2] Members of the Organisation of African Unity. African (Banjul) Charter on Human and Peoples’ Rights. 1981. Available from: https://www.ohchr.org/en/resources/educators/human-rights-education-training/1-african-charter-human-and-peoples-rights-banjul-charter-1981.

[3] Forman L, Ooms G, Chapman A, Friedman E, Waris A, Lamprea E (2013). What could a strengthened right to health bring to the post-2015 health development agenda?: interrogating the role of the minimum core concept in advancing essential global health needs. BMC Int Health Hum Rights.

[4] Hayden P (2012). The human right to health and the struggle for recognition. Rev Int Stud.

[5] Kuruvilla S, Schweitzer J, Bishai D, Chowdhury S, Caramani D, Frost L (2014). Success factors for reducing maternal and child mortality. Bull World Heal Organ.

[6] Bishai DM, Cohen R, Alfonso YN, Adam T, Kuruvilla S, Schweitzer J (2016). Factors contributing to maternal and child mortality reductions in 146 low - and middle - income countries between 1990 and 2010. PLoS One.

[7] Roser M, Ritchie H, Bernadeta D. Child and Infant Mortality. Our world in Data. 2019. Available from: https://ourworldindata.org/child-mortality Cited 2022 Mar 2

[8] Roser M, Ritchie H. Maternal Mortality. 2013. Available from: https://ourworldindata.org/maternal-mortality

[9] United Nations. The Sustainable Development Goals Report 2020. 2020. Available from: https://unstats.un.org/sdgs/report/2020/

[10] Fuest C, Riedel N. Tax evasion, tax avoidance and tax expenditures in developing countries: a review of the literature. Oxford University Centre for Business Taxation; 2009.

[11] WHO Commission on the Social Determinants of Health. Closing the gap in a generation. 2008. Available from: https://www.who.int/health-topics/social-determinants-of-health#tab=tab_1.

[12] Fair tax foundation. Fair Tax Mark accreditation process. Available from: https://fairtaxmark.net/why-get-the-mark/the-process/. Cited 2022 Jan 10

[13] United Nations General Assembly. Addis Ababa Action Agenda of the Third International Conference on Financing for Development (Addis Ababa Action Agenda) A/RES/69/313. 2015; Available from: https://www.un.org/ga/search/view_doc.asp?symbol=A/RES/69/313&Lang=E

[14] International Chamber of Commerce. Position Paper - Tax and the United Nations Sustainable Development Goals. 2015;(Icc):1–8. Available from: https://cdn.iccwbo.org/content/uploads/sites/3/2018/02/icc-position-paper-on-tax-and-the-un-sdgs.pdf

[15] The Tax Justice Network Team. The State of Tax Justice 2020: tax justice in the time of COVID-19. 2020. Available from: https://www.fes.de/en/shaping-a-just-world/climate-change-energy-and-environment/article-on-climate-change-energy-and-environment/the-state-of-tax-justice-2020-tax-justice-in-the-time-of-covid.

[16] International Bar Association. Tax Abuses, Poverty and Human Rights - A report of the International Bar Association’s Human Rights Institute Task Force on Illicit Financial Flows, Poverty and Human Rights. 2013. Available from: http://www.ibanet.org/Human_Rights_Institute/TaskForce_IllicitFinancialFlows_Poverty_HumanRights.aspx

[17] Fisher J (2014). Fairer shores: Tax havens, tax avoidance, and corporate social responsibility. Bost Univ Law Rev.

[18] Sikka P (2010). Smoke and Mirrors: Corporate Social Responsibility and Tax Avoidance. Account Forum.

[19] OECD. Glossary of Tax Terms. OECD Cent Tax Policy Adm. 2020;1–29. Available from: https://www.oecd.org/ctp/glossaryoftaxterms.htm

[20] The Tax Justice Network Team (2020). Methodological note : State of Tax Justice.

[21] United Nations Department of Economic and Social Affairs, United Nations, Nations U. The Sustainable Development Goals. 2021. Available from: https://sdgs.un.org/goals Cited 2021 Jun 23

[22] Rosen MA. Energy Sustainability with a Focus on Environmental Perspectives. Earth Syst Environ. 2021;5(2):217–30 10.1007/s41748-021-00217-6 10.1007/s41748-021-00217-6 PMC8060340 34723079

[23] United Nations - Addis Ababa Action Agenda. Financing for Development; 2015. Available from: https://www.un.org/development/desa/financing/document/addis-ababa-action-agenda.

[24] Reeves A, Gourtsoyannis Y, Basu S, McCoy D, McKee M, Stuckler D. Financing universal health coverage— Effects of alternative tax structures on public health systems: Cross-national modelling in 89 low-income and middle-income countries. Lancet. 2015;386:274–80. Available from: https://www.ncbi.nlm.nih.gov/pmc/articles/PMC4513966/10.1016/S0140-6736(15)60574-8 PMC4513966 25982041

[25] Baldacci E, Clements B, Gupta S, Cui Q (2008). Social spending, human capital, and growth in developing countries. World Dev.

[26] Waris A. Financing Africa. 1st ed. Bamenda: Langaa RPCIG; 2019. 10.2307/j.ctvx07801.

[27] Pirlot A. A legal analysis of the mutual interactions between the UN Sustainable Development Goals (SDGs) & taxation. 2019. Available from: https://papers.ssrn.com/sol3/papers.cfm?abstract_id=3467544.

[28] Mazzucato M, Roll K, Aeron Thomas D, McPherson M. Lorca de Urarte MA. The Biscay Model: Aligning Tax Policy with the United Nations Sustainable Development Goals; 2021. p. 1–5. Available from: https://www.ucl.ac.uk/bartlett/public-purpose/publications/2021/may/biscay-model-aligning-tax-policy-united-nations-sustainable-development-goals.

[29] Moore M. Obstacles to Increasing Tax Revenues in Low Income Countries. SSRN Electronic Journal. 2014. Available from: http://papers.ssrn.com/sol3/papers.cfm?abstract_id=2436437

[30] African Union (AU), African Tax Administration Forum (ATAF), Organisation for Economic Development (OECD), Development Centre. Revenue Statistics in Africa 1990–2019. 2021. Available from: https://www.oecd.org/ctp/revenue-statistics-in-africa-2617653x.htm Cited 2022 Oct 4

[31] Ligomeka WY. Tax Revenue Mobilisation in Malawi. 2019. Available from: http://sro.sussex.ac.uk/id/eprint/86257/

[32] Durst MC. Taxing Multinational Business in Lower-Income Countries: Economics, Politics and Social Responsibility. 2019. Available from: https://opendocs.ids.ac.uk/opendocs/bitstream/handle/123456789/14336/Durst_Book_Final.pdf?sequence=1&isAllowed=y Cited 2019 Apr 11

[33] Bebbington J, Unerman J. Achieving the United Nations Sustainable Development Goals: An enabling role for accounting research. Accounting, Audit Account J [Internet]. 2018;31(1):2–24. Available from: https://www.emerald.com/insight/content/doi/10.1108/AAAJ-05-2017-2929/full/pdf?casa_token=i0SgH8g-XmEAAAAA:RAvMTFeJwW2ZRsGSxWY3FhuAb4HUlfq4ZeRxxl0F_lcQkiC2ta2_Icb6N-VLKU56u1AqQwT7rbQCU5UhFlyWPI7SC3bIRHua3YD59DKEpn5hUWbmnPM

[34] Hall S, Lopez M, Murray S, O’Hare B. Government revenue, quality of governance and child and maternal survival. Appl Econ Lett. 2021;29:16, 1541–46. 10.1080/13504851.2021.1963408.

[35] O’Hare B, Hall SG. The impact of government revenue on the achievement of the sustainable development goals and the amplification potential of good governance. Cent Eur J Econ Model Econom. 2022;14:109–29. Available from: 10.24425/cejeme.2022.142627.

[36] Gaspar V, Jaramillo L, Wingender P (2016). Tax Capacity and Growth: Is there a Tipping Point?. IMF Work Pap.

[37] Hall SG, O’Hare B. WIDER Working Paper 2022/102-A model to explain the impact of government revenue on the quality of governance and the SDGs. 2022 [cited 2022 Sep 23] 10.35188/UNU-WIDER/2022/236-2

[38] Baskaran T, Bigsten A (2013). Fiscal Capacity and the Quality of Government in Sub-Saharan Africa. World Dev.

[39] Carroll AB, Shabana KM, Scherer RW. The Business Case for Corporate Social Responsibility: A Review of Concepts, Research and Practicei jmr_275 85..106. 2010;

[40] Okpara JO, Idowu SO. Corporate social responsibility. Challenges, opportunities and strategies for 21st Century leaders. The Routledge Companion to Non-Market Strategy. 2013. 315 p. Available from: http://link.springer.com/10.1007/978-3-642-40975-2

[41] Asgary N, Li G (2016). Corporate Social Responsibility: Its Economic Impact and Link to the Bullwhip Effect. J Bus Ethics.

[42] Visser W (2014). The Age of Responsibility: CSR 2.0 and the New DNA of Business. J Law Gov.

[43] Christensen J, Murphy R. The Social Irresponsibility of Corporate Tax Avoidance: Taking CSR to the bottom line. Dev 2004 473. 2004 Aug 31 [cited 2022 Jan 30];47(3):37–44. Available from: https://link.springer.com/article/10.1057/palgrave.development.1100066

[44] Vodafone Group PLC. Vodafone. 2022 [cited 2022 Oct 10]. Available from: https://www.vodafone.com/

[45] Whalley J, Curwen P. Managing tax by organizational means: the case of Vodafone. Public Money Manag [Internet]. 2014;34(5):371–8. Available from: https://www.tandfonline.com/doi/full/10.1080/09540962.2014.945809

[46] Sikka P (2012). The tax avoidance industry. Radic Stat.

[47] Vodafone Group PLC. and our total contribution to public finances [Internet]. Available from: https://www.vodafone.com/about-vodafone/reporting-centre/tax-and-economic-contribution

[48] O’Hare B, Makuta I. An analysis of the potential for achieving the fourth millennium development goal in SSA with domestic resources. Global Health. 2015;11(1). 10.1186/s12992-015-0092-1 PMC4362663 25885642

[49] O’Hare BAM, Lopez MJ, Mazimbe B, Murray S, Spencer N, Torrie C, et al. Tax abuse—The potential for the Sustainable Development Goals. PLOS Glob Public Heal. 2022;2(2):e0000119 10.1371/journal.pgph.0000119 10.1371/journal.pgph.0000119 PMC10021515 36962275

[50] Hall S, Illian J, Makuta I, Mcnabb K, Murray S, Hare BAMO, et al. Government revenue and child and maternal mortality. Open Econ Rev. 2021;32:213–29. Available from: 10.1007/s11079-020-09597-0.

[51] O’Hare B, Hall S, Murray S. The Government Revenue and Development Estimations (GRADE). 2020. Available from: http://medicine.st-andrews.ac.uk/grade/

[52] Hall S, O’Hare B, Murray S. Mendeley Dataset: The impact of government revenue on the determinants of child health and the amplification potential of good governance. 2020;1.

[53] United Nations University. The Government Revenue Dataset. 2020. Available from: https://www.wider.unu.edu/project/grd-–-government-revenue-dataset

[54] The World Bank. World Development Indicators. 2021. Available from: https://databank.worldbank.org/source/world-development-indicators Cited 2022 May 3

[55] Kaufmann D, Kraay A, Mastruzzi M. The worldwide governance indicators methodology and analytical issues. Dev Res Group, World Bank; 2010. Available: https://openknowledge.worldbank.org/bitstream/handle/10986/3913/WPS5430.pdf?sequence=1&isAllowed=y.

[56] Vodafone Group PLC. Tax and our total contribution to public finances. 2015. Available from: https://www.vodafone.com/about-vodafone/reporting-centre/tax-and-economic-contribution Cited 2022 Mar 22

[57] Vodafone Group. Taxation and our total economic contribution to public finances 2018. Vodafone Group PLC; 2018. Cited 2022 Mar 22. Available from https://www.vodafone.com/about-vodafone/reporting-centre/tax-and-economic-contribution.

[58] Vodafone Group PLC. Taxation and our total economic contribution to public finances Taxation and our total economic contribution to public finances. 2016. Available from: https://www.vodafone.com/about-vodafone/reporting-centre/tax-and-economic-contribution Cited 2022 Mar 22

[59] Vodafone Group PLC. Taxation and our total economic contribution to public finances 2016–17 Taxation and our total economic contribution to public finances. 2017. Available from: https://www.vodafone.com/about-vodafone/reporting-centre/tax-and-economic-contribution Cited 2022 Mar 22

[60] Vodafone Group PLC. Tax and our total contribution to public finances. 2014. Available from: https://www.vodafone.com/about-vodafone/reporting-centre/tax-and-economic-contribution Cited 2022 Mar 22

[61] Vodafone Group. Available from: https://www.vodafone.com/about-vodafone/reporting-centre/tax-and-economic-contribution.

[62] HMRC. HMRC Annual Report and Accounts 2017 to 2018 - Executive Summary. 2018 [cited 2022 Feb 9]. Available from: https://www.gov.uk/government/publications/hmrc-annual-report-and-accounts-2017-to-2018/hmrc-annual-report-and-accounts-2017-to-2018-executive-summary.

[63] Hall S, Illian J, Makuta I, McNabb K, Murray S, Haider Ali Zaidi S, et al. Government revenue and child and maternal mortality. Open Econ Rev. 2020;32:213–29. Available from: 10.1007/s11079-020-09597-0.

[64] Cobham A, Janský P. Measuring misalignment: the location of US multinationals’ economic activity versus the location of their profits. Dev Policy Rev. 2019;37(1):91–110.

[65] Barnett ML. Stakeholder influence capacity and the variability of financial returns to corporate social responsibility. Acad Manag Rev. 2007;32(3):794–816. Available from: https://journals.aom.org/doi/abs/10.5465/AMR.2007.25275520. Cited 2022 Dec 8.

[66] Oxfam. Raising the bar: rethinking the role of business in the Sustainable Development Goals. 2017. Available from: https://www.oxfam.org/en/research/raising-bar-rethinking-role-business-sustainable-development-goals.

[67] Kovermann J, Velte P. The impact of corporate governance on corporate tax avoidance—A literature review. J Int Accounting, Audit Tax. 2019;36.

[68] Organisation for Economic Development (OECD). Base erosion and profit shifting - OECD BEPS. Available from: https://www.oecd.org/tax/beps/. Cited 2022 Dec 8.

[69] Tomukorpi K. Corporate tax avoidance: is paying a fair share of taxes a social responsibility? 2020. Available from: https://www.theseus.fi/handle/10024/339171.

[70] Friedman M. The social responsibility of business is to increase its profits. In: Zimmerli WC, Richter K, Holzinger M, editors. Corporate Ethics and Corporate Governance: Springer; 2007 [cited 2022 Dec 8]. Available from: https://link.springer.com/content/pdf/10.1007/978-3-540-70818-6.pdf?pdf=button.

[71] Mao CW. Effect of corporate social responsibility on corporate tax avoidance: evidence from a matching approach. Qual Quant. 2019;53(1):49–67. Available from: https://www.proquest.com/openview/fdf349f6ce7b47126c809833e3e4d930/1?pq-origsite=gscholaramp;cbl=54128.

[72] Gulzar MA, Cherian J, Sial MS, Badulescu A, Thu PA, Badulescu D, et al. Does corporate social responsibility influence corporate tax avoidance of Chinese listed companies? Sustain. 2018;10(12). Available from: https://www.mdpi.com/2071-1050/10/12/4549.

[73] Huseynov F, Klamm BK. Tax avoidance, tax management and corporate social responsibility. J Corp Financ. 2012;18(4):804–27. Available from: https://www.sciencedirect.com/science/article/abs/pii/S0929119912000569?via%3Dihub. Cited 2022 Jan 30.

[74] Lanis R, Richardson G (2012). Corporate social responsibili ty and tax aggressiveness: an empirical analysis. J Account Public Policy.

[75] The Tax Justice Network. Tax justice & human rights: the 4 Rs and the realisation of rights. 2021 [cited 2021 Jul 9]. Available from: https://taxjustice.net/2021/07/06/tax-abuse-and-human-rights/.

[76] Matos P. ESG and responsible institutional investing around the world: a critical review. 2020. https://www.cfainstitute.org/en/research/foundation/2020/esg-and-responsible-institutional-investing.

[77] Pope S, Kim J. Where, when, and who: corporate social responsibility and brand value—A global panel study. Bus Soc. 2021;61. 10.1177/0007650321101931579.

[78] Hanlon M, Slemrod J (2009). What does tax aggressiveness signal? Evidence from stock price reactions to news about tax shelter involvement. J Public Econ.

[79] Hardeck I, Harden JW, Upton DR (2021). Consumer reactions to tax avoidance: evidence from the United States and Germany. J Bus Ethics.

[80] Alexander RM (2013). Tax transparency. Bus Horiz.

[81] Bauer R, Ruof T, Smeets P (2021). Get Real! Individuals prefer more sustainable investments. Rev Financ Stud.

[82] Principles of Responsible Investment. What are the principles for responsible investment? PRI; 2021. https://www.unpri.org/about-us/what-are-the-principles-for-responsible-investment.

[83] What does stakeholder capitalism mean for investors? The Investors Forum and London Business School. 2022. Available from: https://www.investorforum.org.uk/wpcontent/uploads/securepdfs/2022/05/Stakeholder_Capitalism-report.pdf. Cited 6/12/22.

[84] The High Level Panel on International Financial Accountability Transparency and Integrity. Financial integrity for sustainable development. 2021. Available from: https://www.factipanel.org/about.

[85] Xu R. Financial integrity for sustainable development-implementation of FACTI panel recommendations in Africa. 2022;1–38. Available from: https://www.un.org/osaa/sites/www.un.org.osaa/files/financial_integrity_for_sustainable_development_in_africa_en.pdf.

[86] Office of the United Nations High Commissioner for Human Rights. Guiding principles on business and human rights guiding principles on business and human rights. New York and Geneva; 2011. Available from: https://www.ohchr.org/en/publications/reference-publications/guiding-principles-business-and-human-rights.

[87] Committee on Economic Social and Cultural Rights (CESCR). General Comment No. 24 on State Obligations under the International Covenant on Economic, Social and Cultural Rights in the Context of Business Activities. E/C.12/GC/24. 2017.

[88] Tax Justice Network. The State of Tax Justice 2021. 2021. Available from: https://taxjustice.net/wp-content/uploads/2021/11/State_of_Tax_Justice_Report_2021_ENGLISH.pdf.

[89] Shaxson N. Tackling tax havens. Finance Dev. 2019:6–10. Available from: https://www.imf.org/external/pubs/ft/fandd/2019/09/pdf/tackling-global-tax-havens-shaxon.pdf.

[90] The Tax Justice Network and GRADE. Submission to the Human Rights Council Universal Periodic Review (Fourth Cycle) United Kingdom of Great Britain & Northern. 2022. p. 1–12. Available from: https://medicine.st-andrews.ac.uk/grade/wp-content/uploads/sites/39/2022/12/Submission-to-Human-Rights-Council-Universal-Periodic-Review-Fourth-Cycle-UK-NI.pdf.

[91] Antonio Alonso J. Two major gaps in global governance: international tax cooperation and sovereign debt crisis resolution. J Glob Dev. 2019;9(2):20180016. 10.1515/jgd-2018-0016.

[92] Global Alliance for Tax Justice. Press release: governments approve proposal for International Tax Cooperation at United Nations - Global Tax Justice. 2022 [cited 2022 Dec 7]. Available from: https://globaltaxjustice.org/news/press-release-governments-approve-proposal-for-international-tax-cooperation-at-united-nations/.

